# Supplementary material for: The efficacy of receptor tyrosine kinase EphA2 autophosphorylation increases with EphA2 oligomer size
Source: J Biol Chem. 2022 Aug 13;298(10):102370. doi: 10.1016/j.jbc.2022.102370 (PMC9512837; doi:10.1016/j.jbc.2022.102370)
Supplement: Supporting information [file mmc1.pdf]

SUPPLEMENT

The efficacy of EphA2 tyrosine phosphorylation increases with EphA2 oligomer size

Elmer Zapata-Mercado[1], Gabriel Biener[2], Daniel M. McKenzie[1], William C. Wimley[4], Elena B. Pasquale[5], Valerica Raicu*[2,3] and Kalina Hristova*[1]

[1]Department of Materials Science and Engineering, Johns Hopkins University, 3400 Charles Street, Baltimore, MD 21218;

[2]Department of Physics, University of Wisconsin, Milwaukee, 3135 N. Maryland Ave, WI 53211;

[3]Department of Biological Sciences, University of Wisconsin, Milwaukee, 3209 N. Maryland Ave, WI 53211

[4]Tulane University School of Medicine, Department of Biochemistry and Molecular Biology, New Orleans, LA;

[5]Sanford Burnham Prebys Medical Discovery Institute, 10901 North Torrey Road, La Jolla, CA 92037;

Whole Membrane	Intercept	Slope	P-value	Corr (R^2)
ε_{Mean}				
β_{lig}	0.84 (0.38, 1.29)	-0.02 (-0.07, 0.04)	>0.05	0.14
**E_{top} pY588**	-0.12 (-0.42, 0.18)	0.09 (0.04, 0.14)	<0.01	0.83
**E_{top} $pAKT_{inh}$**	1.09 (0.92, 1.26)	-0.02 (-0.04, 0.00)	0.05	0.63
**EC_{50} (pY588)/ EC_{50} ($pAKT_{inh}$)**	-1.16 (-9.05, 6.72)	0.87 (-0.37, 2.10)	>0.05	0.49
ε_{Median}				
β_{lig}	0.85 (0.31, 1.39)	-0.03 (-0.12, 0.07)	>0.05	0.12
**E_{top} pY588**	-0.17 (-0.69, 0.34)	0.14 (0.02, 0.25)	0.03	0.65
**E_{top} $pAKT_{inh}$**	1.17 (0.89, 1.34)	-0.03 (-0.07, 0.00)	0.05	0.55
**EC_{50} (pY588)/ EC_{50} ($pAKT_{inh}$)**	-1.64 (-13.22, 9.94)	1.24 (-1.18, 3.66)	>0.05	0.34
ε_{Mode}				
β_{lig}	0.81 (0.11, 1.51)	-0.03 (-0.28, 0.21)	>0.05	0.06
**E_{top} pY588**	0.09 (-0.90, 1.30)	0.20 (-0.35, 0.54)	>0.05	0.06
**E_{top} $pAKT_{inh}$**	1.03 (0.64, 1.42)	-0.03 (-0.17, 0.10)	>0.05	0.10
**EC_{50} (pY588)/ EC_{50} ($pAKT_{inh}$)**	4.99 (-13.15, 23.14)	-0.29 (-6.77, 6.19)	>0.05	0.00

Table S1: Weighted linear regression analyses to determine if there is a statistically significant correlation between the EphA2 signaling characteristics and the mean, median, and mode of the molecular brightness log-normal distributions for the whole membrane. The intercept (and 95% CI) and the slope (and 95% CI) for each linear regression are shown, as calculated from the values reported in Table 2. Correlation coefficients were determined from the fits. P-values were determined by comparing the slope to the null hypothesis of zero slope using a one sample t-test.

Puncta	μ	σ	Mean	Median	Mode
**ephrinA1-Fc**	2.42 ± 0.04	0.69 ± 0.03	14.25 ± 0.59	11.21 ± 0.40	6.94 ± 0.38
**m-ephrinA1**	2.08 ± 0.03	0.63 ± 0.02	9.78 ± 0.28	8.03 ± 0.20	5.41 ± 0.20
**dimer 2**	2.91 ± 0.08	0.84 ± 0.06	26.01 ± 2.68	18.32 ± 1.52	9.09 ± 1.22
**dimer 5**	2.96 ± 0.05	0.54 ± 0.02	22.37 ± 0.61	19.31 ± 0.47	14.38 ± 0.49
**dimer 8**	2.18 ± 0.01	0.44 ± 0.01	9.76 ± 0.10	8.87 ± 0.08	7.32 ± 0.09
**monomer 10**	2.31 ± 0.04	0.70 ± 0.03	12.84 ± 0.53	10.07 ± 0.35	6.19 ± 0.33
**YSA-bio**	2.20 ± 0.01	0.56 ± 0.01	10.56 ± 0.17	9.03 ± 0.13	6.59 ± 0.14

Table S2: Parameters of the molecular brightness log-normal distributions obtained from the high intensity puncta analysis. μ and σ are the two best-fit parameters of the respective ln(brightness) normal distributions (see equation (2)). The mean, median and mode of the log-normal distributions are calculated according to equations (3), (4) and (5), respectively.

Puncta	Intercept	Slope	P-value	Corr (R^2)
ε_{Mean}				
β_{lig}	0.53 (0.38, 0.67)	0 (0, 0.02)	>0.05	0.70
E_{top} pY588	0.52 (-0.12, 1.16)	0 (-0.03, 0.03)	>0.05	0.02
E_{top} $pAKT_{inh}$	0.98 (0.74, 1.21)	0 (-0.02, 0.01)	>0.05	0.09
EC_{50} (pY588)/ EC_{50} ($pAKT_{inh}$)	1.32 (-6.78, 9.42)	0.13 (-0.48, 0.74)	>0.05	0.40
ε_{Median}				
β_{lig}	0.56 (0.40, 0.72)	0 (-0.01, 0.02)	>0.05	0.47
E_{top} pY588	0.49 (-0.15, 1.12)	0 (-0.04, 0.05)	>0.05	0.04
E_{top} $pAKT_{inh}$	0.94 (0.74, 1.14)	0 (-0.02, 0.01)	>0.05	0.01
EC_{50} (pY588)/ EC_{50} ($pAKT_{inh}$)	2.37 (-6.15, 10.9)	0.06 (-0.76, 0.88)	>0.05	0.18
ε_{Mode}				
β_{lig}	0.59 (0.40, 0.78)	0.01 (-0.02, 0.03)	>0.05	0.24
E_{top} pY588	0.19 (-0.38, 0.75)	0.04 (-0.02, 0.10)	>0.05	0.60
E_{top} $pAKT_{inh}$	0.98 (0.67, 1.29)	0 (-0.04, 0.02)	>0.05	0.13
EC_{50} (pY588)/ EC_{50} ($pAKT_{inh}$)	-1.73 (-8.75, 5.28)	0.89 (-0.22, 1.99)	>0.05	0.68

Table S3: Weighted linear regression analyses to determine if there is a statistically significant correlation between the EphA2 signaling characteristics and the mean, median, and mode of the molecular brightness log-normal distributions for the high-intensity puncta. The intercept (and 95% CI) and the slope (and 95% CI) for each linear regression are shown for the values reported in Table 4. Correlation coefficients were determined from the fits. P-values were determined by comparing the SE_{slope} to the null hypothesis of zero slope using a one sample t-test.

Whole Membrane		Puncta	
	**Mean**		**Mean**
**no ligand**	2.52 ± 0.02	**no ligand**	N/A
**monomer 10**	5.22 ± 0.05	**dimer 8**	9.76 ± 0.10
**m-ephrinA1**	5.60 ± 0.03	**m-ephrinA1**	9.78 ± 0.28
**dimer 2**	6.12 ± 0.09	**YSA-bio**	10.56 ± 0.17
**YSA-bio**	6.20 ± 0.05	**monomer 10**	12.84 ± 0.53
**ephrinA1-Fc**	7.17 ± 0.09	**ephrinA1-Fc**	14.25 ± 0.59
**dimer 5**	10.02 ± 0.16	**dimer 5**	22.37 ± 0.61
**dimer 8**	11.66 ± 0.16	**dimer 2**	26.01 ± 2.68

Table S4: Rank order of peptide mean brightness is different for whole membranes and puncta.

Supplemental Figures

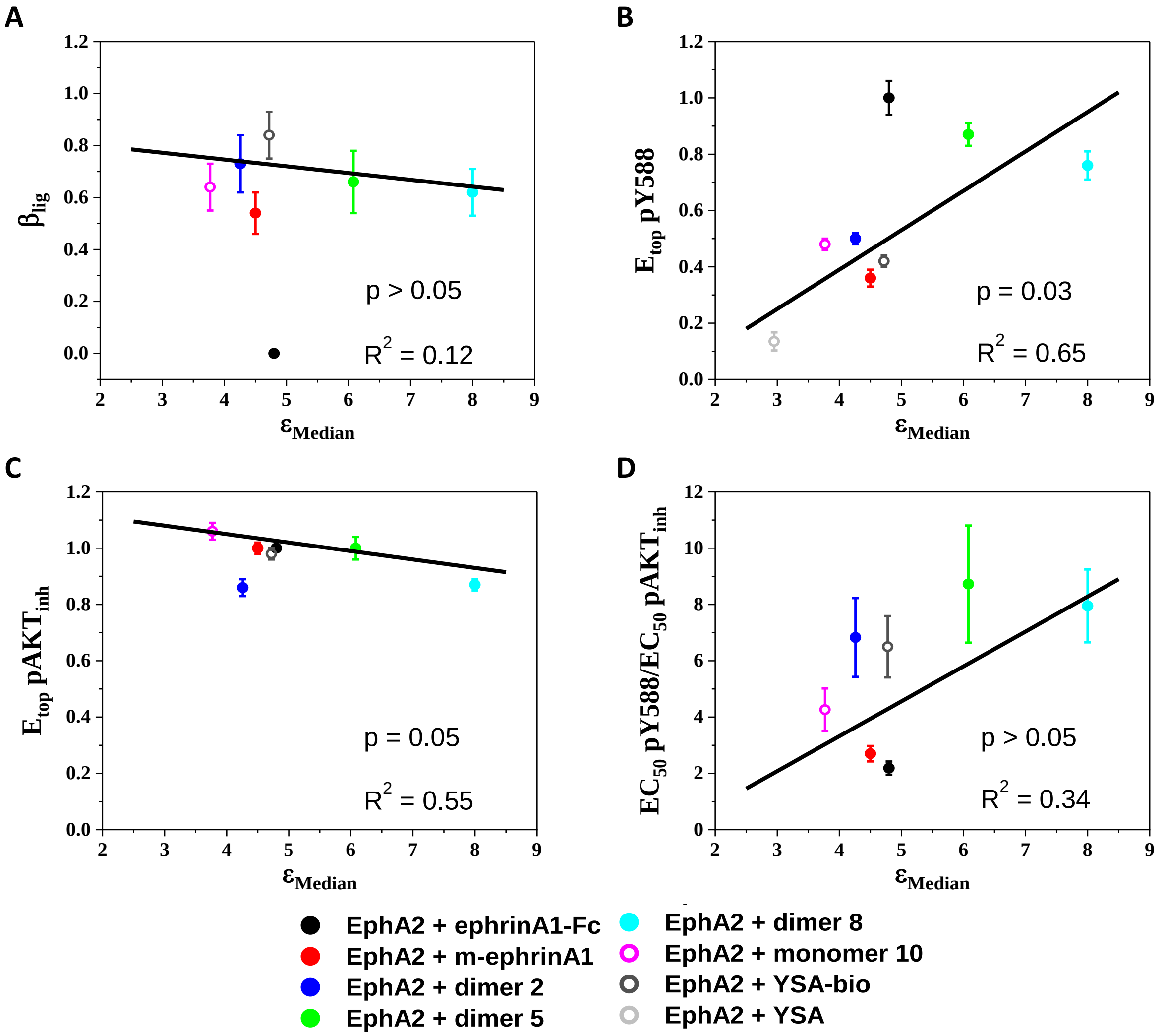

Figure S1: Correlation between EphA2 signaling parameters and the **medians** of the molecular brightness log-normal distributions obtained from FIF analysis of whole membranes. (A) Ligand bias coefficients versus medians, when ephrinA1-Fc was used as the reference ligand. (B) Ligand-specific EphA2 Y588 phosphorylation efficacies, normalized to the value obtained with the reference ligand ephrinA1-Fc, versus the medians. (C) Ligand-specific AKT inhibition efficacies, normalized to the value obtained with the reference ligand ephrinA1-Fc, versus medians. (D) Ligand-specific ratios of Y588 phosphorylation to AKT inhibition potencies versus medians. Data points: averages and standard errors from (1). Lines: linear fits, excluding ephrinA1-Fc. EphA2 + YSA only shown in panel (B).

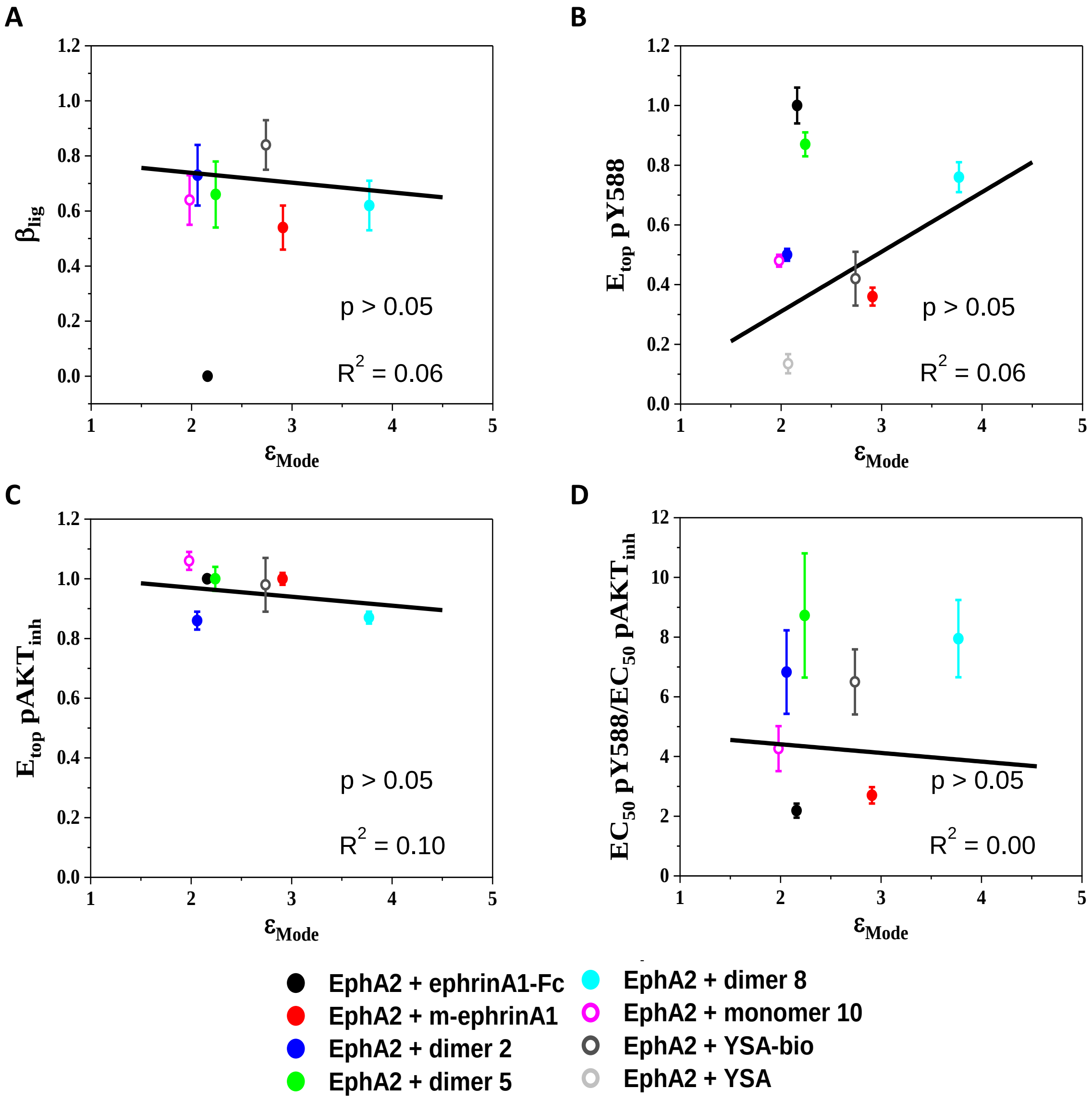

Figure S2: Correlation between EphA2 signaling parameters and the **modes** of the molecular brightness log-normal distributions obtained from FIF analysis of whole membranes. (A) Ligand bias coefficients versus modes, when ephrinA1-Fc was used as the reference ligand. (B) Ligand-specific EphA2 Y588 phosphorylation efficacies, normalized to the value obtained with the reference ligand ephrinA1-Fc, versus the modes. (C) Ligand-specific AKT inhibition efficacies, normalized to the value obtained with the reference ligand ephrinA1-Fc, versus modes. (D) Ligand-specific ratios of Y588 phosphorylation to AKT inhibition potencies versus modes. Data points: averages and standard errors from (1). Lines: linear fits, excluding ephrinA1-Fc. EphA2 + YSA only shown in panel (B).

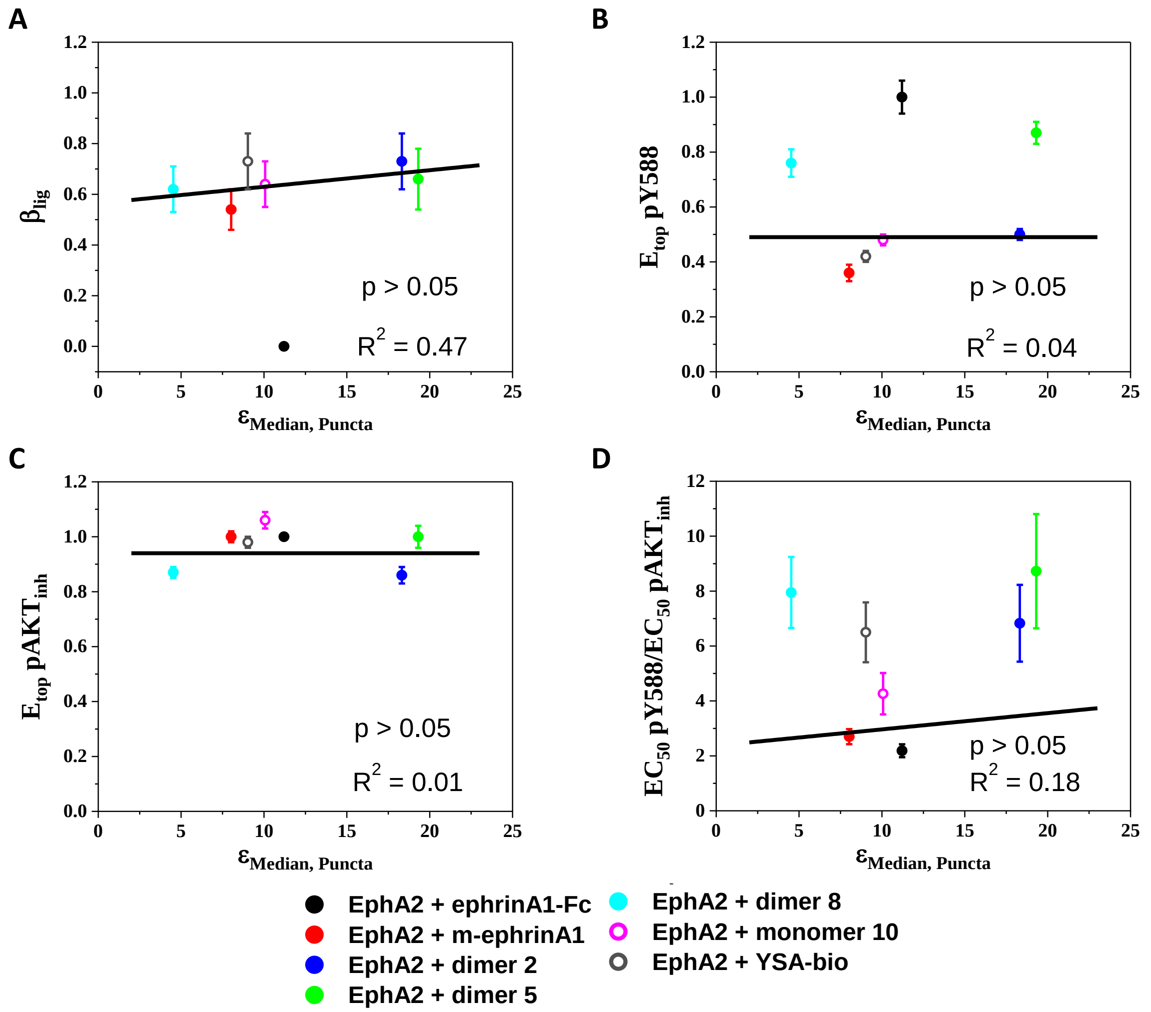

Figure S3: Correlation between EphA2 signaling parameters and the **medians** of the molecular brightness log-normal distributions obtained from FIF analysis of the high-intensity puncta. (A) Ligand bias coefficients versus medians, when ephrinA1-Fc was used as the reference ligand. (B) Ligand-specific EphA2 Y588 phosphorylation efficacies, normalized to the value obtained with the reference ligand ephrinA1-Fc, versus the medians. (C) Ligand-specific AKT inhibition efficacies, normalized to the value obtained with the reference ligand ephrinA1-Fc, versus medians. (D) Ligand-specific ratios of Y588 phosphorylation to AKT inhibition potencies versus medians. Data points: averages and standard errors from(1). Lines: linear fits, excluding ephrinA1-Fc. Shown are only data for ligands which induce puncta formation.

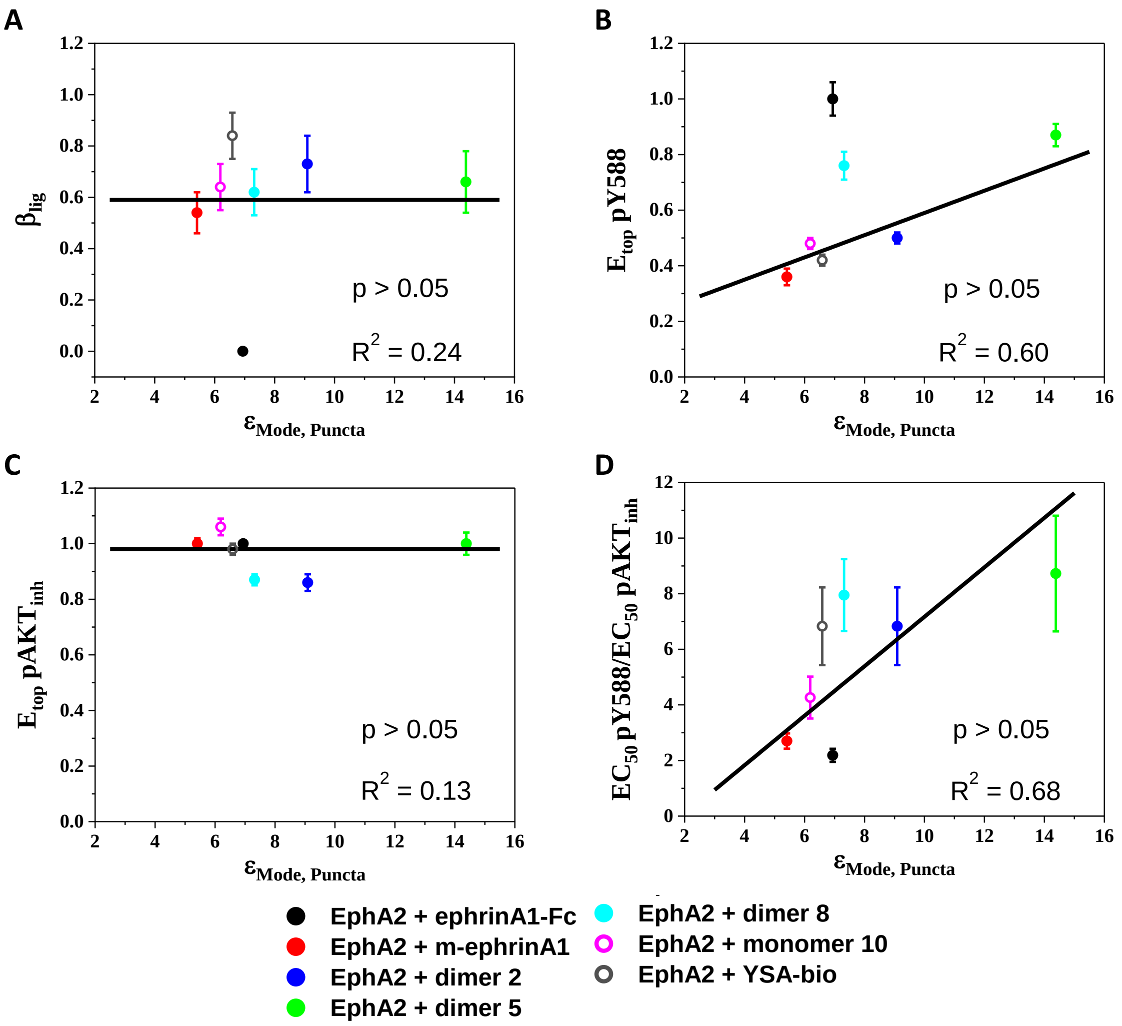

Figure S4: Correlation between EphA2 signaling parameters and the **modes** of the molecular brightness log-normal distributions obtained from FIF analysis of the high-intensity puncta. (A) Ligand bias coefficients versus modes, when ephrinA1-Fc was used as the reference ligand. (B) Ligand-specific EphA2 Y588 phosphorylation efficacies, normalized to the value obtained with the reference ligand ephrinA1-Fc, versus the modes. (C) Ligand-specific AKT inhibition efficacies, normalized to the value obtained with the reference ligand ephrinA1-Fc, versus modes. (D) Ligand-specific ratios of Y588 phosphorylation to AKT inhibition potencies versus modes. Data points: averages and standard errors from (1). Lines: linear fits, excluding ephrinA1-Fc. Shown are only data for ligands which induce puncta formation.

1. Gomez-Soler, M., Gehring, M. P., Lechtenberg, B. C., Zapata-Mercado, E., Ruelos, A., Matsumoto, M. W., Hristova, K., and Pasquale, E. B. (2022) Ligands with different dimeric configurations potently activate the EphA2 receptor and reveal its potential for biased signaling. *iScience* **25**, 103870
